# The location of the axon initial segment affects the bandwidth of spike initiation dynamics

**DOI:** 10.1371/journal.pcbi.1008087

**Published:** 2020-07-23

**Authors:** Christophe Verbist, Michael G. Müller, Huibert D. Mansvelder, Robert Legenstein, Michele Giugliano

**Affiliations:** 1 Molecular, Cellular, and Network Excitability Laboratory, Institute Born-Bunge and Department of Biomedical Sciences, Universiteit Antwerpen, Wilrijk, Belgium; 2 Institute of Theoretical Computer Science, Graz University of Technology, Graz, Austria; 3 Department of Integrative Neurophysiology, Amsterdam Neuroscience, Center for Neurogenomics and Cognitive Research (CNCR), Vrije Universiteit Amsterdam, Amsterdam, The Netherlands; 4 International School of Advanced Studies, Neuroscience Area, Trieste, Italy; University of Toronto at Scarborough, CANADA

## Abstract

The dynamics and the sharp onset of action potential (AP) generation have recently been the subject of intense experimental and theoretical investigations. According to the resistive coupling theory, an electrotonic interplay between the site of AP initiation in the axon and the somato-dendritic load determines the AP waveform. This phenomenon not only alters the shape of APs recorded at the soma, but also determines the dynamics of excitability across a variety of time scales. Supporting this statement, here we generalize a previous numerical study and extend it to the quantification of the input-output gain of the neuronal dynamical response. We consider three classes of multicompartmental mathematical models, ranging from ball-and-stick simplified descriptions of neuronal excitability to 3D-reconstructed biophysical models of excitatory neurons of rodent and human cortical tissue. For each model, we demonstrate that increasing the distance between the axonal site of AP initiation and the soma markedly increases the bandwidth of neuronal response properties. We finally consider the Liquid State Machine paradigm, exploring the impact of altering the site of AP initiation at the level of a neuronal population, and demonstrate that an optimal distance exists to boost the computational performance of the network in a simple classification task.

## Author summary

The neurons in the brain encode information through electrical impulses. The performance of a cell in terms of its ability to process and transfer information downstream thus depends heavily on the machinery of initiation of these impulses. In this work, we consider both the cell morphology and the biophysical properties of impulse initiation as the primary parameters that influence information processing in single neurons, as well as in networks. We specifically analyze the location of nerve impulse initiation along the cell’s axon and the way the neuron transfers incoming information. By using single-cell models of various complexity as well as network models, we conclude that information processing is sensitive to the geometrical details of impulse initiation.

## Introduction

The dynamics of AP initiation and its underlying time-scales have been themes of intense investigation in rodent and human cortical neurons, both experimentally [1–8] and theoretically [6,9–12]. Investigations have focused particularly on the shape of the somatic AP, its rapidity at onset, and on its underlying biophysics [1,6,10,13]. In fact, early numerical and theoretical studies on single-compartmental models of spike-initiation [9,14] suggested a strong causal relationship between the rapidity of the AP at its onset and the dynamics of the instantaneous firing rate. The latter ultimately determine the encoding and tracking properties of neurons and networks of rapid components in their input [8]. Indeed, neurons with rapid APs are better at tracking very fast temporal modulations of their inputs [5,7], than neurons with smooth AP waveforms.

The proposed biophysical bases, underlying rapid APs, have thus been linked to ion channel cooperativity [1,15], axo-somatic backpropagation [13], and to the electrotonic coupling of dendro-somatic compartments to the site of AP initiation in the axon [10,11]. Recently, Brette and collaborators further explored how the specific location of the AP initiation in the axon (i.e. the axon initial segment, AIS) could affect the AP rapidity at its onset [12] and reviewed the diversity of AIS and axon location [16]. Specifically, they demonstrated *in silico* that increasing the AIS distance from the soma makes the AP somatic waveform sharper than for proximal AIS locations [12].

Therefore, we expect that altering the AIS location during development or upon its activity-dependent plasticity [17,18] should also influence the bandwidth of the neuron input-output firing response properties, thus ultimately changing its computational performance.

In this work, we numerically characterized how a non-stationary input is capable of destabilizing instantaneously the (otherwise stationary) output firing frequency of a model neuron, active on average at 5 spike/s. Mimicking previous experimental protocols [2–4,7,8], we applied a weak oscillation on the top of a current-clamp stimulus and observed how the instantaneous firing rate of the model oscillates around 5 spike/s (e.g. 5 ± 3 spike/s). Similarly to the experimental studies, faster input oscillations (e.g. 10–50–100–500–1000 cycle/s) lead to a faster oscillation of the instantaneous firing rate, although with stronger amplitude attenuation. The study of this attenuation, as a function of the oscillation frequency, quantifies the linear transfer gain of the model neuron and constitutes a minimal description of the dynamics of its AP initiation. We then systematically examined the linear transfer gain in three classes of conductance-based multicompartmental model neurons. For each model, we altered the somatic distance of the AIS by controlling the density of voltage-gated sodium and potassium ion channels along the axon and estimated the (low-pass) cut-off harmonic frequency (COF) of the resulting linear transfer gain. We specifically included in our study a large class of 3D-reconstructed models of cortical neurons, using the detailed biophysical models database recently released by the Blue Brain Project [19]. Furthermore, we investigated the influence of the AP onset dynamics and response bandwidth on the computational power of a neuronal network. To this end, we used the Liquid State Machine (LSM) paradigm, a standard model for generic computations in cortical microcircuits [20].

## Results

**Fig 1:**
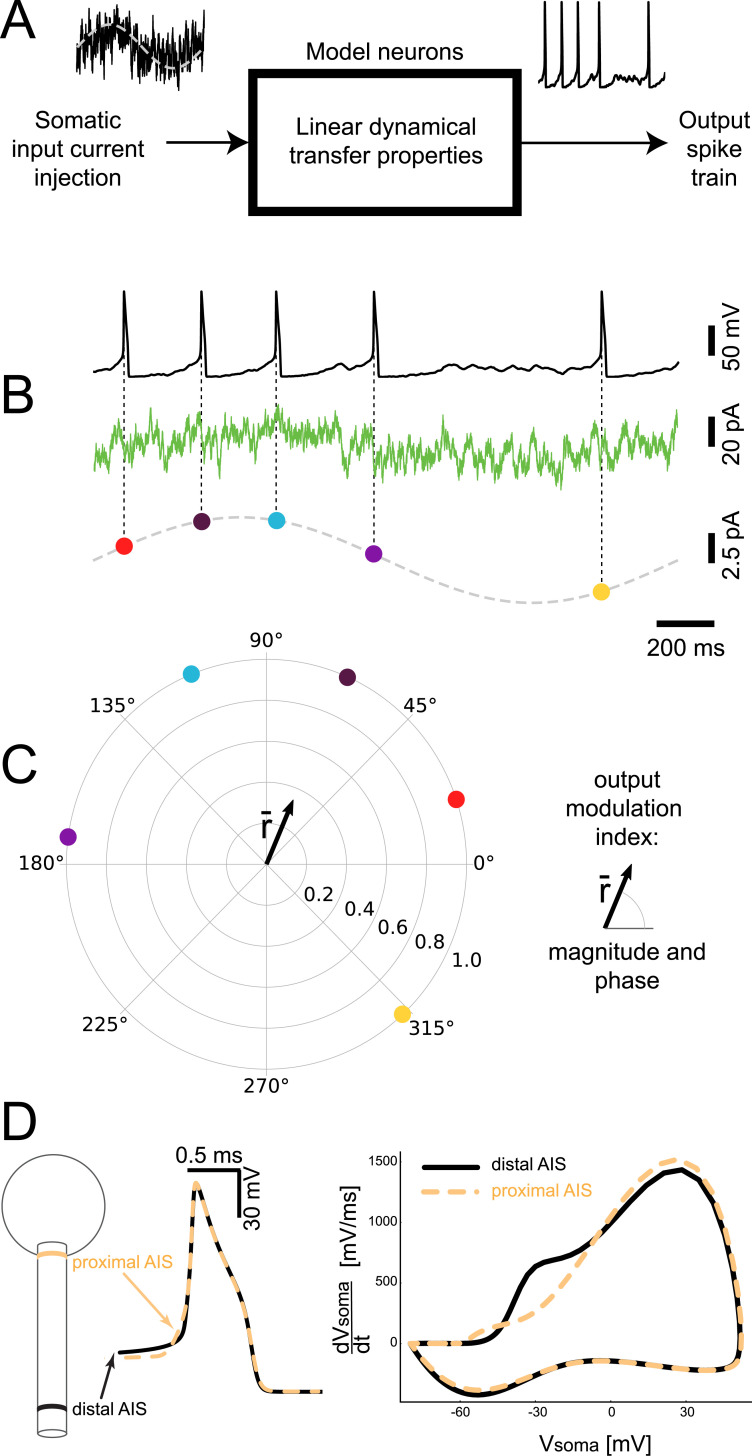
**Linear dynamical transfer properties of multicompartmental model neurons.** We studied the dynamics of AP initiation in neuronal models, by estimating the temporal modulation of the instantaneous firing probability, in response to the somatic injection of a noisy current (B, green trace). The offset of this current (A-B, dashed grey trace) was weakly modulated over time at a harmonic frequency *f*. With Circular Statistics methods, we referred the time of each AP to the corresponding phase of the input oscillation. Then we regarded in the complex plane each AP as a vector with unitary length (C, filled colored markers) (C). We finally estimated the magnitude and phase of the vector sum $\bar{r}$ (C), averaging together tens of thousands of APs and thus resulting in a vector with length lower than 1. We systematically explored magnitude and phase for a broad range of values of *f* (i.e. 10–1000 cycle/s). As the soma-AIS distance increased, the somatic AP waveform varied and became steeper (D).

We studied the dynamics of the excitability in multicompartmental neuron models with increasing complexity. We aimed to directly reproduce, *in silico*, an experimental protocol previously applied *in vitro*, thus enabling the comparison with existing electrophysiological data. Instead of characterizing the conventional (stationary) spiking response to DC current pulses, we measured the instantaneous firing rate while injecting weak sinusoidal stimuli with a variety of harmonic frequencies *f*, as well as fluctuating noisy waveforms into the soma of each model neuron (Fig 1A). We referred the timing of each AP to the phase of the input sine (Fig 1B) and applied circular statistics (see the Methods) to quantify, for each harmonic frequency *f*, the magnitude and the phase of the instantaneous firing rate (Fig 1C). These quantities describe the transfer gain associated with the dynamic response of each model (Fig 1A), as in the previous experiments [2]. This allowed us to quantify how a temporal modulation of the input current influenced the instantaneous output firing rate of neurons, firing on average at *5* spike/s (see the Methods).

### The AIS location alters neuronal responsiveness

**Fig 2:**
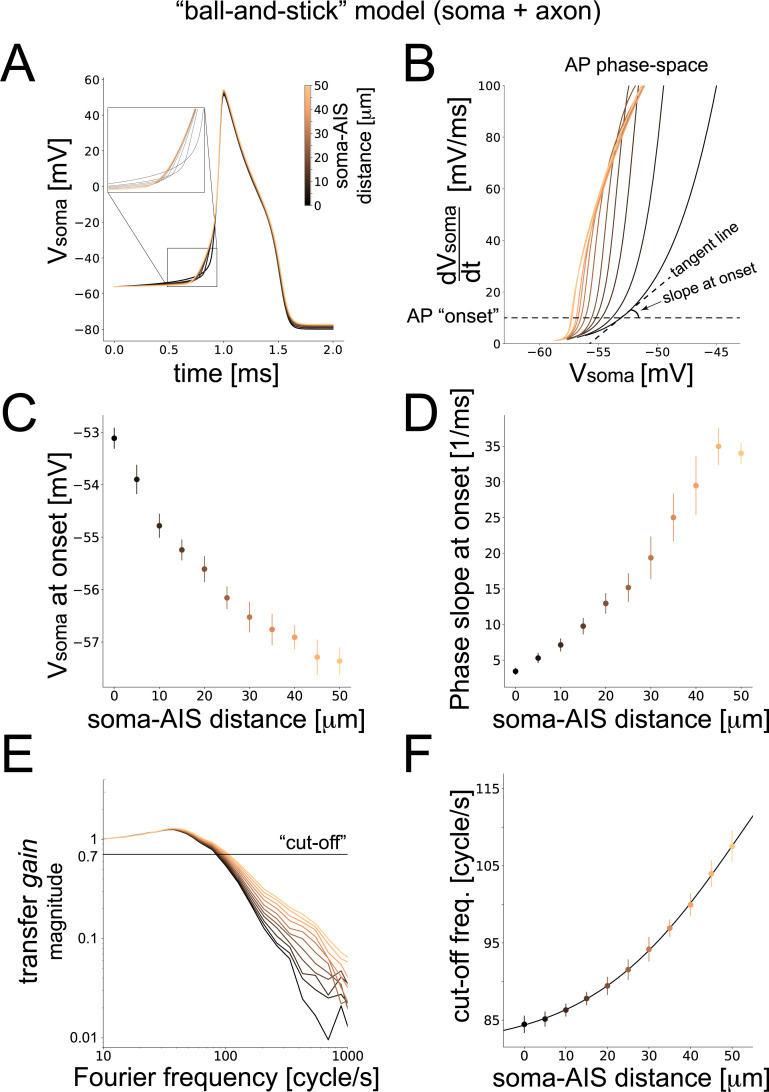
**Performance of the “ball-and-stick” neuron model.** Color-coding across panels reflects the soma-AIS distances, with darker colors used for more proximal AIS locations and brighter colors for more distal AIS locations. The somatic AP waveform was examined in time (A) and in the phase-space, plotting the derivative of the potential *versus* the potential (B). We conventionally set the AP “onset” to 10mV/ms (dashed horizontal black line), deriving the value of AP threshold (i.e. the potential at the onset) (C) and the AP rapidity (i.e. the phase slope at onset) (D). The magnitude of the dynamical transfer gain of the model was estimated as in Fig 1 and plotted in the Fourier domain, across increasing soma-AIS distances, normalized to its value at 1 cycle/s (E). The “cut-off” frequency, defined as the harmonic frequency corresponding to a 30% attenuation of the magnitude, was then studied against the soma-AIS distance (D) and fitted by a logistic function. Error bars (C-F) represent the standard deviation over 100 independent repeated simulations.

For each model neuron under consideration, we systematically varied the location of the AIS with respect to the soma and quantified the dynamics of neuronal responses, extending the scope of a recent study [12]. We started with a minimal model, composed of a soma and a multicompartmental axon (“ball and stick”, BAS). While relocating the AIS hardly altered the model’s input resistance (i.e. by ~0.01%), we found that it greatly affected the steepness of somatic action potentials at their onset (Fig 2A and 2B), confirming the previous study. Specifically, we found a 7-fold increase in the slope of the action potential trajectory at onset (Fig 2D), corresponding to an increasing distance between the AIS and the soma of up to 50 μm. We also observed a ~4 mV hyperpolarization of the membrane potential at onset (Fig 2C), conventionally identifying the AP “threshold” potential, for the same range of AIS distances from the soma. Indicating an increase in cell excitability, a similar hyperpolarization has already been predicted by the critical resistive coupling theory [10] in terms of a smaller sodium-channel current eliciting an AP and resulting from the weakening of the intensity of the axo-somatic resistive current [12].

Similar to real neurons, as we probed the response of the BAS model to temporally modulated noisy waveforms (Fig 1), we observed a low-pass filter behavior in the Fourier domain and characterized for large input harmonic frequency *f* by a 1/*f* ^α^ power law. The value of its exponent α decreased from 1.82 to 1.03 almost linearly with an increasing AIS distance from the soma (Fig 2E). The low pass filter gain profile was normalized with its value at *f* = 1 *cycle/s* and then quantified in terms of a conventional cutoff frequency (COF). Such a COF describes the value of *f* where the transfer gain attenuates down to 70% of its normalized amplitude (Fig 2E). In our numerical study, we observed a ~30% increase in the COF, ranging from ~85 cycle/s to ~110 cycle/s (Fig 2F) as the AIS moved away from the soma up to 50 μm. Note how the increase in the COF was accompanied by a change in the slope of the curves, exclusively for large Fourier frequencies (Fig 2E).

### Multicompartmental neuron models

We repeated the same analysis for a family of 3D reconstructed multicompartmental models of rat neocortical neurons, released by the Blue Brain Project (BBP). Restricting our focus to excitatory cells only, we first report the results observed for a L5 thick-tufted pyramidal cell (TTPC). We augmented these models with a functional multicompartmental axon, identical in geometry and biophysical properties to the one used in the BAS model. Note that in the original multicompartmental description of the cell’s morphology by the BBP, the axon was also simplified to a short “stick” version.

**Fig 3:**
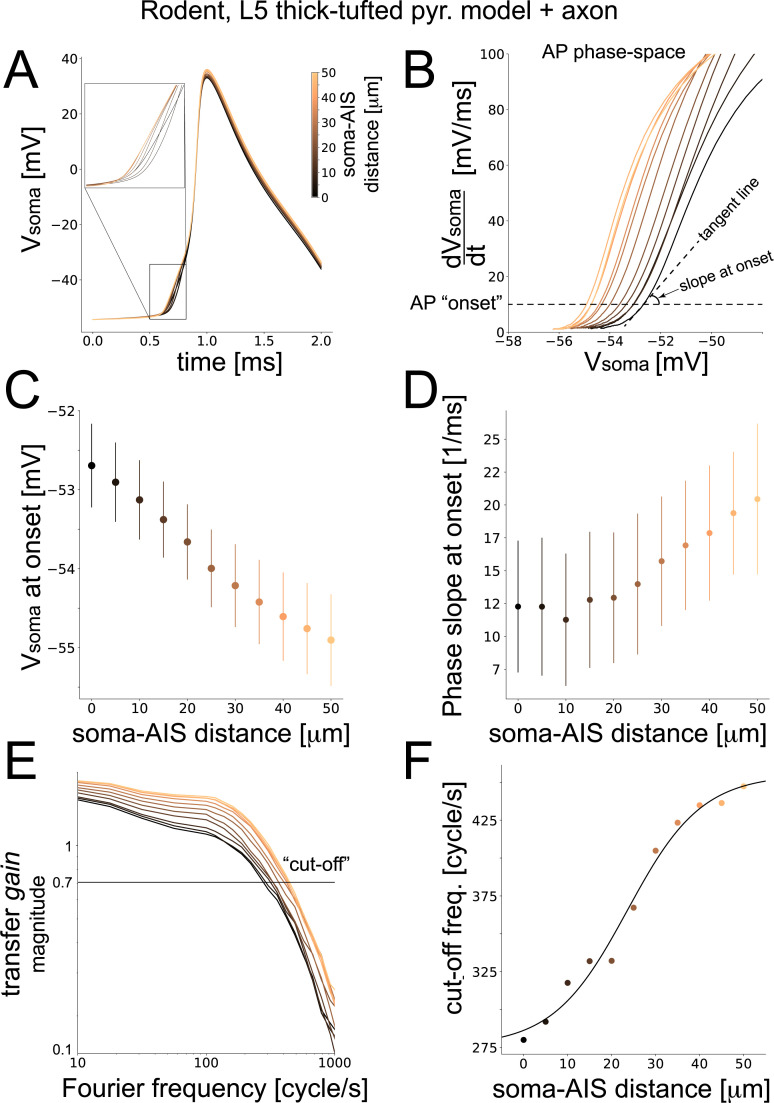
**Performance of a rat cortical pyramidal neuron model.** We repeated the analysis of Fig 2 for a model of rat neocortical layer 5 pyramidal cells. As in the “ball-and-stick” model, when the AIS moved away from the soma (A), the somatic AP became steeper (A), while its threshold potential decreased (B,C) and its rapidity at onset increased (B,D). The magnitude of the dynamical transfer gain of the model was plotted in the Fourier domain, across increasing soma-AIS distances, normalized to its value at 1 cycle/s (E). The “cut-off” frequency was then studied against the soma-AIS distance (D) and fitted by a logistic function. Color coding as in Fig 2 and error bars (C-F) representing the standard deviation over 100 independent repeated simulations.

The somatic AP slope at its onset increased with the AIS—soma distance, although markedly less (~2-fold) than in the BAS model (Fig 3A–3D). The progressive hyperpolarization of the membrane potential at onset was similar in all cases (~ 3 mV difference; Fig 3C) and similarly corresponding to an excitability increase predicted by the critical resistive theory [10,12].

When examined in the Fourier domain, the magnitude of the transfer gain was quantitatively very similar to experimental reports [2,6,8] with a COF well above *f =* 100 cycle/s. Interestingly, its low-pass profile featured roughly the same slope at large harmonic frequencies *f*, regardless of the AIS location (Fig 3E). Notably, increasing the AIS—soma distance caused a 55% increase in the value of the COF, ranging from ~275 cycle/s to ~450 cycle/s and effectively altering the overall bandwidth of the neuronal response [7].

Encouraged by the agreement with the previous experimental data and intrigued by the significant modulation of neuronal bandwidth by the AIS location, we further studied the same phenomenon in all other 65 model cells of the BBP database, representative of 13 distinct types of excitatory neurons (i.e. 65 = 13 * 5 instances of each type) [19]. As for the TTPC, we augmented each of the 65 models with a multicompartmental axon, identical to the cases discussed so far. Not only did we confirm similar effects of the AIS location on the transfer bandwidth in all other excitatory cells, but we could also rank the response width and sensitivity of each model.

**Fig 4:**
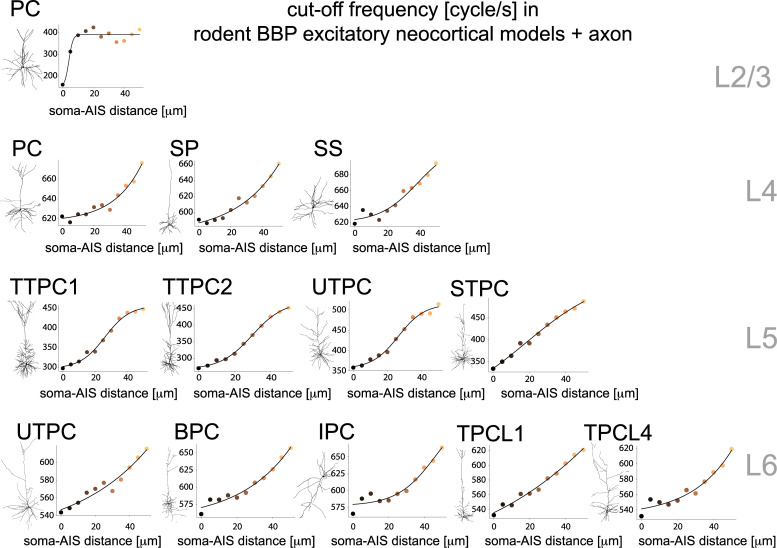
**Performance of various excitatory rat cortical neuron models.** We repeated the analysis of Figs 2 and 3, focusing on the “cut-off” frequency sensitivity to the soma-AIS distance (see Figs 2F and 3F) of all the 13 excitatory neuron models, as released by the Blue Brain Project. Each panel refers to a distinct cell type across cortical layers 2/3, 4, 5, and 6, and is representative of Pyramidal Cells (PC), Star Pyramidal cells (SP), Spiny Stellate neurons (SS), Thick-Tufted Pyramidal Cells (TTPC), Untufted Pyramidal Cells (UTPC), Slender Tufted Pyramidal Cells (STPC), Pyramidal Cell with Bipolar apical-like dendrites (BPC), Pyramidal Cell with Inverted apical-like dendrites (IPC), Tufted Pyramidal Cell with apical dendrites terminating in Layer 1 (TPCL1), and Tufted Pyramidal Cell with apical dendrites terminating in Layer 4 (TPCL4). The continuous traces represent logistic functions whose parameters (Table 1) have been best fitted to the simulation results. Color coding as in Fig 2.

According to our simulations (Fig 4), the neuronal cells with the broadest bandwidth of their transfer gain are those located in layer 4, followed in ranking by those in layer 6, layer 5, and finally in layer 2/3. Fig 4 summarizes how the COF varies with the increasing soma–AIS distance, accompanying data points (colored markers) by sigmoidal best fit functions (black continuous traces). However, when studying the sensitivity to the AIS location of the bandwidth, the previous ranking reversed with layer 2/3 cells exceeding all the other layers (Fig 4; see Table 1 and the curve slope *k* parameter). Regardless of the cell type, layer 4 cells displayed the weakest sensitivity to the AIS location of their bandwidth, with a change of ~10–12%. Similarly, L6 cells had a modest increase in their COFs, quantified as ~14–17%, for increasing values of the AIS—soma distance. L5 had a stronger dependency, with an increase up to 60%. We thus observed an extremely high sensitivity to AIS location for L2/3 pyramidal cells, with up to a ~150% increase of the COF (in the range 200–400 cycles/s). In addition, over all 65 models tested, the slope of the transfer *gain* for high harmonic frequencies *f* did not vary substantially upon moving away the AIS from the soma, as observed for the TTPC (Fig 3E).

Summarizing the sensitivity of the COF on the AIS—soma distance, we provide below the best-fit parameters of a logistic function (see the Methods), indicating the layer and the cell type:

**Table 1:** Best-fit parameters. For each different excitatory model of the BBP database, the best fit parameters of a logistic function (Eq 4) of Fig 4 are represented in this table.

	****a****	****k****	****d**_**0**_**	****b****
L23—PC	242.86	0.747	4.02	148.73
L4—PC	3339.00	0.056	121.02	616.93
L4—SP	2261.71	0.038	134.15	573.59
L4—SS	98.24	0.086	38.65	620.23
L5—TTPC1	309.86	0.043	18.14	237.51
L5—TTPC2	160.00	0.129	26.12	296.38
L5—UTPC	203.68	0.114	29.74	266.72
L5—STPC	158.90	0.130	26.45	355.61
L6—UTPC	2875.42	0.035	141.00	552.11
L6—BPC	153.73	0.091	47.16	577.21
L6—IPC	471.09	0.023	85.16	478.35
L6—TPCL1	2762.32	0.045	125.18	532.61
L6—TPCL4	3235.00	0.023	196.43	515.90

As a further validation experiment, we also inspected the phase of the transfer gain for one of the models (L6 TTPC-L4), as we increased the AIS—soma distance (Figure A in S1 Text). As expected from the experimental data [2,8], we found that the relationship between the phase and the AIS distance could be fitted by a straight line for large harmonic frequencies *f* [14]. By definition, a linear dependency on *f* in the Fourier domain corresponds to the presence of a time delay in the time domain. We could therefore verify that the farther the AIS is from the soma, the longer the propagation delay is of the AP generated at the AIS and recorded at the soma.

**Fig 5:**
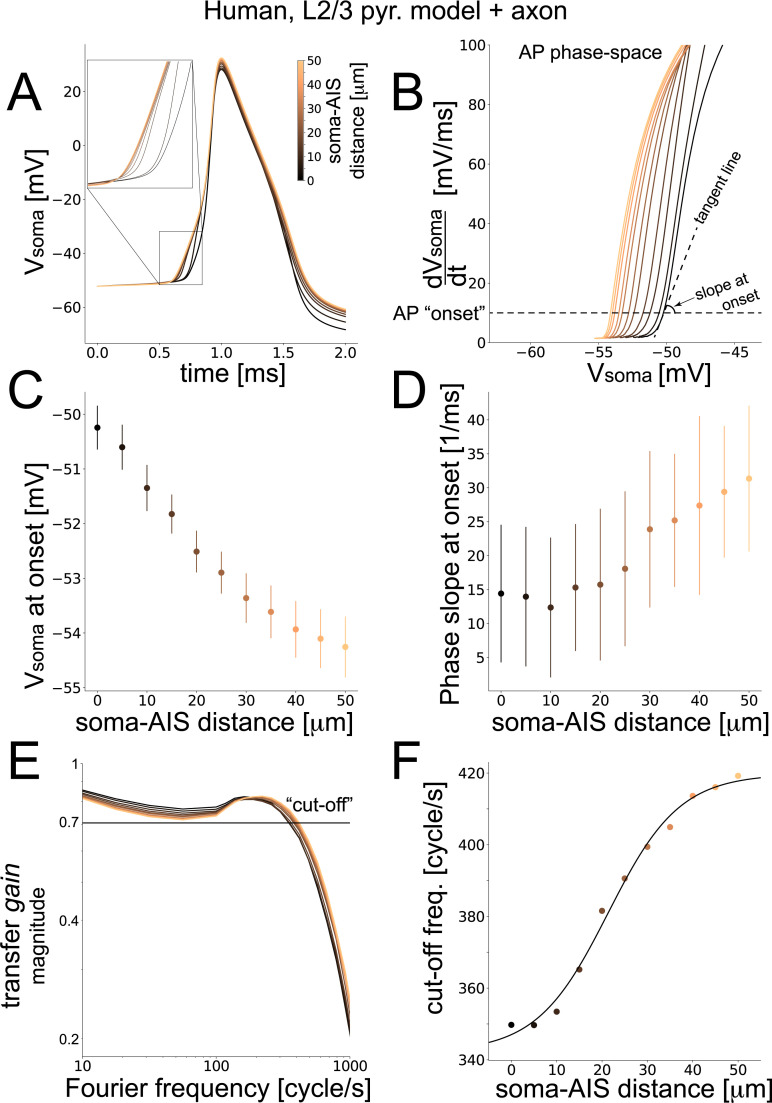
**Performance of a human cortical neuron model.** We repeated the analysis of Figs 2 and 3 for a multicompartmental model of human neocortical layer 2/3 pyramidal cells. When the AIS moved away from the soma (A), the somatic AP became steeper (A), while its threshold potential decreased (B,C) and its rapidity at onset increased (B,D). The magnitude of the dynamical transfer gain of the model was plotted in the Fourier domain, across increasing soma-AIS distances, normalized to its value at 1 cycle/s (E). The “cut-off” frequency was then studied against the soma-AIS distance (D) and fitted by a logistic function. Color coding as in Fig 2 and error bars (C-F) representing the standard deviation over 100 independent repeated simulations.

Finally, as larger dendritic trees have been shown to contribute to the dynamical transfer properties of neurons [11], we studied the dynamical response of a model reconstructed from the human neocortical tissue (HUM, Fig 5). This model was available from a previous study [21] and featured a morphology considerably larger than any rat cortical cell. In the HUM model, we observed only a modest sensitivity (~2 fold) to the AIS position of the AP slope at the onset, while the increase in excitability by a hyperpolarization of the somatic AP threshold voltage was comparable to the other models. When examining the transfer gain, only a ~20% increase in the COF was observed (Fig 5F). Note also how the increase in the COF was not accompanied by any change in the slope of the curves for large Fourier harmonic frequencies *f* (Fig 5E), as opposed to Fig 2E.

Concluding this section, Table 2 summarizes for the BAS, the L5 TTPC pyramidal BBP, and the HUM models, the sensitivity of the COF on the AIS—soma distance *d* as the best-fit parameters of a logistic function (see the Methods).

**Table 2:** Best fit parameters across models. The best-fit parameters for the logistic functions in Figs 2, 3F and 5F are represented in this table.

	****a****	****k****	****d**_**0**_**	****b****
BAS	57.69	0.0541	53.32	81.3
TTPC BBP	180.9	0.1167	23.47	247.1
HUM	77.56	0.1271	21.35	342.2

### Network simulations

**Fig 6:**
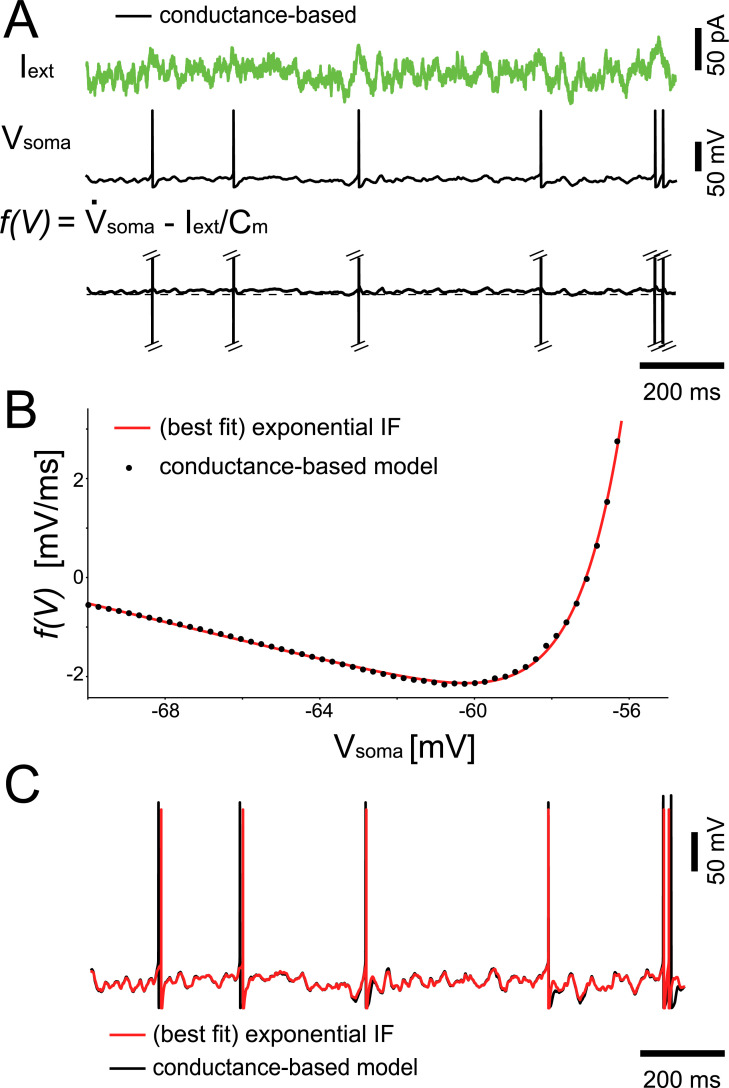
**Reduction to a point neuron model.** We tuned the parameters of an exponential Integrate-and-Fire (eIF) point neuron model to optimally match the membrane potential of the ball-and-stick model, in response to the same noisy input current. (A) The voltage-dependent AP initiation current was isolated by subtraction, (B) best fitted to the current-voltage relationship of the eIF, and (C) shown to adequately capture both the timing of individual APs and the trajectory of subthreshold membrane potential.

**Fig 7:**
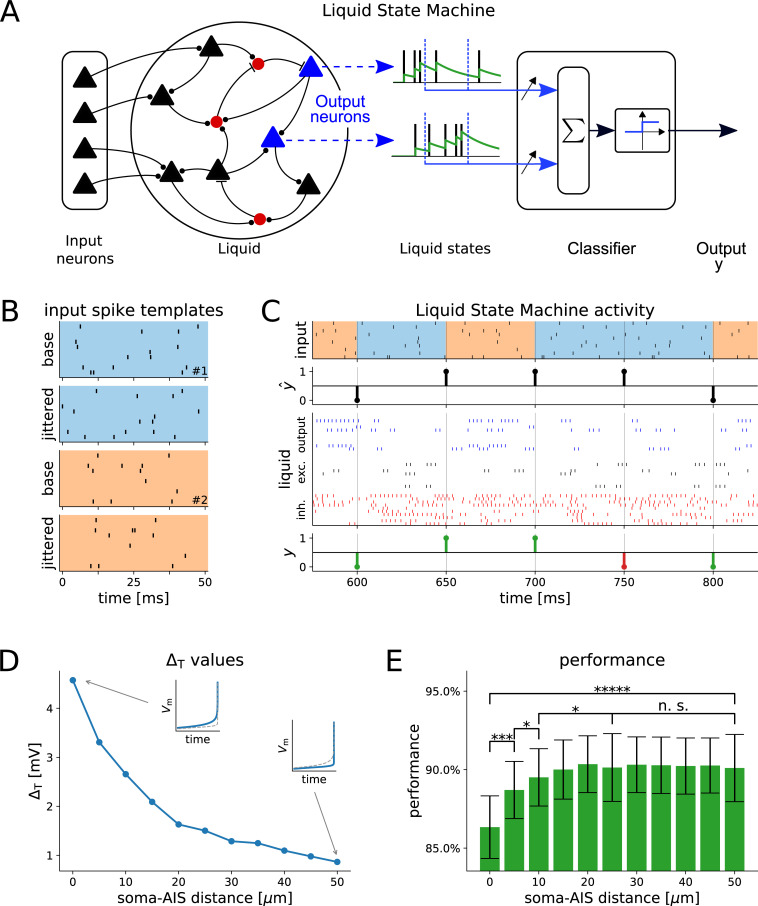
**Liquid-state machine computations.** In order to test the functional impact of the AIS location and the bandwidth of the transfer gain, we simulated a network of exponential Integrate-and-Fire (eIF) units using parameters fit to models with different AIS locations (see Fig 6 and Table 3). (A) The input was fed to a pool of recurrently connected neurons (black and blue: excitatory, red: inhibitory). Neurons were connected randomly through dynamic synapses. The filtered spikes (liquid states) of a subset of excitatory neurons (output neurons, blue), was used as input to a linear classifier. (B) The network input consisted of jittered versions of two base spike templates. (C) The classifier was trained to compute a XOR of the last two shown templates (top) using the spikes of the output neurons (blue) in the liquid (middle). As performance criterion we recorded how often the readout response *y* (bottom) matched the target output $\hat{y}$ (correct outputs are shown in green, incorrect outputs in red) for the parameters for different AIS locations (e.g. the AP slope Δ_T_). (D) The fitted Δ_T_ values are shown versus the soma-AIS distance. The insets show the change of the slope at the AP onset from the first to the last AIS position. (E) As we varied the AIS locations, the Liquid State Machine performance improved in the classification task. The effect was significant for the first two distance increments (50 runs, Wilcoxon rank-sum test, * = p < 0.05, ** = p < 0.005, etc.).

Since the bandwidth of AP initiation dynamics ultimately determines the signal transfer properties of large networks [14], we hypothesized that changing the AIS location might to some extent influence the computational properties of a neuronal network. We therefore investigated the influence of the AIS location on the performance of a Liquid State Machine (LSM) model [20]. As all multicompartmental models examined here were computationally expensive, we reduced the BAS model into an equivalent single-compartmental exponential integrate-and-fire (eIF) model [14] (Fig 6), closely following established methods [22,23]. In an LSM, a cortical microcircuit is modelled as a randomly connected network of excitatory and inhibitory integrate-and-fire neurons (the “liquid”, see Fig 7A) with dynamic synaptic transmission [24]. Projection neurons in cortical layers III and V are modelled in the LSM as linear readout neurons, which receive as input filtered spike trains from a random subset of neurons in the liquid. We used the output of the readout neurons as output of the LSM. When inputs are presented to the network via a number of input neurons, the recurrent connections give rise to prolonged reverberating activity in the liquid, whose rich dynamics are used by the linear readout (trained as a supervised classifier) to achieve some desired output behavior. It has been shown that this simple model possesses remarkable computational capabilities as it can approximate any fading memory filter [20].

To determine the computational capabilities of these LSMs, we considered a delayed XOR task (Fig 7B and 7C). This simple task tests two important properties of the network: its short-term memory [25] and its nonlinear processing capabilities, both potentially linked to the AP initiation dynamics. We generated two prototypical input AP patterns lasting 50 ms and using 10 input channels (Fig 7B) and presented a random sequence of jittered versions of these patterns (spike shifts drawn from a Gaussian with zero mean and 5 ms standard deviation). We trained a binary classifier as readout to compute the XOR of the input at the end of each pattern presentation.

We used this setup to study the influence of the AIS location on the performance of the LSM (Fig 7D) by varying the intrinsic parameters used for the neurons within the *liquid*. The systematic reduction of the multicompartmental models into a point neuron [22] resulted in one set of eIF parameters for each AIS–soma distance (see the Methods), allowing us to determine the computational effect of changing the AIS location.

We found that the location of the AIS had a significant impact on the LSM performance (Fig 7E), as the mean classification accuracy increased from 86% when the AIS was located at 0 μm to over 90% when the AIS was pushed far away from the soma. The increase in LSM performance stopped after a distance of 20 μm was reached, revealing a saturating regime. The differences in the mean network accuracy were small but highly significant (see Fig 7E). The parameter sets for different AIS locations differ primarily in the value of Δ_T_, which sets the AP sharpness in the eIF model, but also in other values (see Table 3). One might therefore ask whether Δ_T_, by defining the signal transfer behavior of individual neurons [14], exerts the main influence on the network performance. We repeated the same simulations using the eIF parameters at one of the medium distances, and varied only Δ_T_ in the set {0.5, 2.5, 4.5} mV (roughly the value range for this parameter found by the fitting procedure). The results show a significant correlation between the AP sharpness and the LSM performance (Figure C in S1 Text), which suggests that the observed effect of the AIS location on network performance is mainly driven by the changes of the AP slope at spike onset. We furthermore performed additional experiments with different sets of network parameters (see Methods and Figure C in S1 Text). The results (Figure C in S1 Text) show that the relationship between soma-AIS distance and network performance holds in a number of different scenarios.

**Table 3:** eIF parameter for BAS model. Numerical parameters, resulting from fitting eIF models to the BAS model with a different AIS-soma distance d, are reported in the table.

****AIS-soma d (μm)****	****τ**_**m**_**(ms)****	****E**_**L**_**(mV)****	****V**_**T**_**(mV)****	****Δ**_**T**_**(mV)****
0	3.40	-74.14	-62.34	4.57
5	5.05	-74.04	-61.28	3.31
10	5.93	-74.09	-60.61	2.66
15	6.89	-74.27	-59.90	2.09
20	7.85	-74.51	-59.14	1.63
25	7.60	-74.39	-59.16	1.50
30	7.86	-74.46	-58.95	1.29
35	7.58	-74.36	-59.14	1.25
40	7.70	-74.39	-59.01	1.10
45	7.74	-74.40	-58.96	0.98
50	7.76	-74.40	-58.89	0.87

## Discussion

This work is grounded in two recent theories: 1) how AP initiation determines the dynamic response of a neuron to time-varying inputs [9,14], and 2) how compartmentalization explains the sharpness of somatic AP initiation [10,12].

The first predicts that the transfer gain, linking in the Fourier domain the instantaneous output firing rate of a neuron to its input, depends on two biophysical properties: the AP sharpness and the average firing rate. Intuitively, we may grasp the former by considering an analogy with the operation of “convolution” (or, equivalently, filtering) of a signal. In fact, when a signal is convolved with a “slow/smooth” function, the result appears blurred, smoothed, and lacks the high-frequency content of the original signal, as in a low-pass electronic filter. On the other hand, when the convolution occurs with a “sharper” function (i.e. as sharp and steep as an impulse), the result more faithfully resembles the original signal. Thus, the sharper the “convolution kernel” the broader the transfer bandwidth, so that sharper somatic AP waveforms correspond to broader bandwidth of the input-output responses of a neuron.

The second theory predicts that an electrotonic interaction between the soma and the axon is responsible for the AP initiation. In this case, the inward sodium currents at the axonal site of AP initiation is matched by the axial resistive current, flowing from the axon to the soma and forming a dipole. This alters the sharpness of the somatic AP, as an intrinsic signature of the global current-loop between the soma and the AIS and not a consequence of studying AP waveforms in a distinct site from where it was generated. In particular, Brette and colleagues [12] showed an increase in the sharpness of the AP when moving the AIS away from the soma.

Combining the predictions of the two theories together, we found that as the AIS relocation modifies the AP sharpness, the bandwidth of the transfer gain of the neuron is also altered. We examined this phenomenon in a variety of multicompartmental mathematical models and found that as the location of the AIS is moved away from the soma, the sharpness of the AP increases and the bandwidth increases, allowing an even more reliable transfer of high-frequency information from the input to the output of the neurons.

It is interesting to set our result in the broader context of an activity-dependent form of AIS homeostatic plasticity, recently described *in vitro* for excitatory neurons [18]. It was elegantly shown that a prolonged increase in the neuronal firing rate triggers a distal displacement of the AIS of up to 17 μm from the soma, while a decrease in firing reversed the effect. We speculate that this plasticity could be functionally linked, and possibly even synergistic, to our results. In fact, as a neuron increases its mean firing rate (e.g. from 5 to 10 spike/s), its bandwidth and COF increase accordingly [9,14]. Then, upon the homeostatic displacement of the AIS location, the COF would even further increase, broadening the overall bandwidth of the input-output dynamical transfer properties of the neurons. Perhaps a similar interaction might support the cooperation between functional and structural plasticities, jointly contributing to make the neuron a broader information channel, capable of routing downstream information with increased temporal accuracy.

To the best of our knowledge, our results are also the first to provide evidence of a very good quantitative match between detailed (i.e. BBP and HUM) cortical neuron models and experimental findings regarding their dynamical transfer function [2,5–8]. Moreover, the simulations described in Fig 4 for all 13 types of excitatory neurons allow us to make a prediction of the cells’ dynamical transfer properties across several layers. It will be very interesting to test in experiments whether the performance ranking across cell types is confirmed. These are important elements supporting the validity and good predictive value of the BBP model database, when tested in experimental protocols not included in the original model optimization procedures [19].

Compared to the dynamics observed in the BAS model, the AP trajectory of BBP models always showed a biphasic behavior at its onset, even when the AIS was closest to the soma. This is a consequence of the electrotonic role played by the dendritic tree, which is absent in the BAS model. Indeed, according to the critical resistive coupling theory [10,12], the BBP models’ dendritic arborizations act as a large current sink for the axon, where the AP is generated. Along these lines, HUM and BBP models differed only quantitatively, given the substantially larger dendritic tree in HUM reconstructed morphology, as already discussed [11].

Despite the simplicity of the (short) axonal geometry used here, our conclusions remain true even for longer and more accurate axonal geometries. In fact, augmenting the original axon model with 1 mm long unmyelinated or myelinated geometry extensions affected our results only quantitatively but not qualitatively (Figure B in S1 Text).

Searching for functional significance of the AIS-soma distance sensitivity (described above for different model neurons), we asked whether a broader or narrower neuronal bandwidth leads to an advantage for information processing in networks of neurons [14]. We investigated this question using the LSM paradigm, a generic model for computations in cortical microcircuits [20,26]. As simulations of large networks of neurons are infeasible using the detailed morphological models described above, we reduced these models to exponential integrate-and-fire units. This reduction resulted in one set of neuron parameters per AIS-soma distance, which allowed us to investigate the impact of the AIS location on the computational capabilities of the network. These single-compartmental neuron models of course fail to capture in its entirety the broad complexity of electrophysiological phenomena of multicompartmental models. Nonetheless, their description of neuronal excitability can mimic AP initiation in an effective manner, allowing us to explore network dynamical properties with modest CPU resources.

We used this network to solve a delayed XOR task, which incorporates two essential components of information processing in neural circuits: short-term memory and nonlinear processing capabilities. Generally, the network was able to solve this task quite well, but the results differed for different AIS locations. We found small but highly significant increases in accuracy as we increased the AIS-soma distance. It is intriguing to note that in L5 cortical cells the AIS starts within 5–10 μm from the soma [27], while the average length of the AIS is 25–40 μm, [28] shows that APs are initiated at the distal end of the AIS, which would correspond to a AIS distance of roughly 25–50 μm in our simulations.

To conclude, we have confirmed that the bandwidth of the spike initiation mechanisms is highly sensitive to the location of the AIS along the axon, in simplified models as well as in a family of biophysically accurate cortical model neurons. We have also shown the impact of the AIS location on computation in a network of neurons. In contrast to the highly complex task solved by cortical networks *in vivo*, we considered a rather simple task and used networks of modest size. It is possible that the significant changes in network performance we observed in our setup will be amplified when larger networks with hierarchical structure and model complex tasks are considered.

## Materials and methods

### Conductance-based model neurons

We simulated three types of conductance-based multicompartmental models, running the NEURON simulator [29,30] on a high-performance computer cluster. The first type, referred to as the “ball-and-stick” (BAS) model in the text, had a single-compartmental soma and no dendrites. The second type, referred to as “Blue Brain Project” (BBP) models in the text, featured somata, realistic dendritic trees, and accurate excitable membrane properties, reconstructed from rat somatosensory cortical neurons over 13 distinct electrical classes [19]. BBP models consisted of 65 distinct models, extracted from a subset (i.e. only excitatory cells) of a previously released database (https://bbp.epfl.ch/nmc-portal/downloads). Finally, the third type, referred to as the “human pyramidal neuron” (HUM) in the text, was based on a 3D-reconstructed pyramidal cell from the superficial layers of human temporal cortical tissue, as described previously [21]. HUM was equipped with very basic excitable membrane properties [21], following closely the approach of [11].

Each BAS, BBP, and HUM model was extended to include an identical multicompartmental axon, originating from the soma, with the same geometry and membrane electrical mechanisms. This axon was described as a cylinder, with a diameter of 1 μm and length of 50 μm and was computer simulated as a set of 11 individual compartments, whose axial resistance and specific capacitance were 150 Ωcm and 0.5625 μFcm^2^, respectively. Each compartment included passive and active ionic currents with parameters chosen as in [13], namely fast-inactivating sodium- and delayed-rectifier potassium-currents, as well as voltage-independent mixed “leakage” currents. Briefly, the Nernst’s reversal potentials of these currents were set uniformly to 60 mV, -80 mV, and -60 mV, for sodium, potassium, and leak currents, respectively. The values of the corresponding maximal ionic conductances varied in space from one compartment to the next. Specifically, while the maximal conductance of the leak currents was fixed along the axon to 3.3 10^−5^ pS/μm^2^, the values for sodium- and potassium-currents differed and were markedly higher (i.e., ~100 times) corresponding to one *ad hoc* compartment, chosen to represent the AIS. These values were 88 nS/μm^2^ and 17.6 nS/μm^2^ at the AIS *versus* 0.8 nS/μm^2^ and 0.16 nS/μm^2^ everywhere else along the axon, for sodium- and potassium-currents, respectively. In additional simulations (Figure B, in S1 Text) we increased the length of the axon, adding other 840 compartments, to model an unmyelinated 1 mm long extension. Alternatively, we implemented a myelinated 1 mm long extension of the axon by 42 additional compartments as in [11].

For the three classes of models considered in this work, the spatial proximity of the AIS to the soma could be varied at will by defining the maximal ionic conductances of each axonal compartment to reflect the AIS location. All model details and computer code are publicly available from FigShare (DOI: 10.6084/m9.figshare.12123279).

### Linear Dynamical Transfer Properties: Spike-Train Analysis in the Fourier Domain

In analogy to an experimental protocol adopted *in vitro* for real neurons to probe the dynamics of their neuronal excitability [2,4,6–8] and also used in previous *in silico* investigations [6,11,14], the BAS, BBP, and HUM models were injected at their somata with a fluctuating stimulation current. This current was composed as the superposition of a DC offset, a sine wave with harmonic frequency *f*, and a Gaussian colored noise:

$$I(t)=I_{0}+I_{1}\;sin(2\pi \;F\;t)+s\;I_{n}(t)$$ (1)

where I_n_(t) was the noise term, generated using an iterative expression [31] and representing a realization of an Ornstein-Uhlenbeck stochastic process [32] with zero mean, unitary standard deviation, and autocorrelation time-length τ. The values of the harmonic frequency *f* were chosen from the range of 1 cycle/s to 10’000 cycle/s in each simulation, which lasted 100 s and was repeated 10–100 times. The numerical integration time step of the model equations was set to 0.005 ms, ensuring numerical stability and avoiding any aliasing of the injected sinusoidal waveform.

For each AIS location, we set I_1_ = 0 and adapted the values of I_0_ (i.e. the stimulus’ mean) and s (i.e. the stimulus’ standard deviation) to achieve the same steady-state output mean firing rate r_0_ of 5 spike/s and the same standard deviation of the subthreshold fluctuations of their membrane potential, in the range 5–10 mV. We note that to this aim, the rescaling of I_0_ and s was very minor, corresponding to changes of less than 2% and 0.001%, respectively. Under such a stationary firing regime, for small amplitudes I_1_ (i.e. I_1_ << I_0_) of the input sine wave, the BAS, BBP, and HUM models neurons generated spike trains, whose instantaneous firing rate r(t) linearly reflected the input oscillation and could be described at the (periodic) steady-state as

$$r(t)=r_{0}+r_{1}\;sin(2\pi \;f\;t+\Phi )$$ (2)

where r_1_ and Φ were found to vary as functions of *f*. These quantities represent the magnitude and the phase of the linear dynamical transfer response of the neuron, expressed as a complex number [2,14]. They were directly estimated from the times of occurrence of somatic spikes t_k_ (i.e. k = 1, 2, …, N) by circular statistics [6], in terms of the magnitude and of the angle of a complex quantity:

$$r=\frac{1}{N}\sum_{k=1}^{N}e^{j\;2\pi \;f\;t_{k}}$$ (3)

where j = √-1 is the imaginary unit. Throughout the figures of this paper, we refer to the response modulation index (i.e. r_1_/r_0_) of the model neuron as the *transfer gain*, estimated as twice the magnitude ||**r**||. We normalized this quantity by its value at *f* = 1 cycle/s, so that neuronal transfer gains could be systematically compared across neuron models and conditions.

For a subset of our BBP model simulations, we also examined the phase Φ that we estimated as the angle of **r**, studying how it changed with *f*. As reported elsewhere for real neurons and in conductance-based models [2,8,14], Φ(*f*) is best described by an additional decreasing linear trend for large values of *f*. Accordingly, the slope of the best-fit straight line to the profile of Φ(*f*) for large values of *f* allowed us to quantify the corresponding propagation delay δt in the time domain.

### Action Potential Trajectory and Transfer Gain profile

AP waveforms were recorded from the somatic compartments in all simulated neuron models, in response to noisy stimulation alone (i.e. setting I_1_ = 0) and under the same steady-state firing regime as already described. The AP trajectory was then examined both in the time domain *versus* V_soma_(t) and in the *phase space* dV_soma_/dt *versus* V_soma_, averaging successive APs over a period of 40 s (i.e. about 200 APs, at 5 spike/s). The analysis in the *phase space* was restricted for the range of values corresponding to the AP initiation (i.e. -70 to 50 mV) [1]. The AP *onset* was conventionally defined as the moment in time during the upstroke AP trajectory when the rate of membrane potential increase exceeded 10 mV/ms [1]. The AP *phase slope at onset* was then defined as the rapidity of the AP trajectory in the phase space at the onset (i.e. the slope of the tangent line to the AP curve at 10 mV/ms).

With regards to the normalized transfer gain, its (low-pass) *cut-off* Fourier harmonic frequency was measured operatively by employing the common definition used in electronic filter analysis as well as in previous works [14]. Corresponding to the “half power” frequency of a filter, the *cut-off* is the value of the harmonic frequency *f* corresponding to a 30% attenuation of the gain (i.e. a value of ~-3 dB). When studied systematically for an increasing soma-AIS distance d, the dependence of the COF on *d* was also summarized by optimally fitting the four parameters of the logistic function:

$$COF(d)=\frac{a}{1+e^{-k(d-d_{0})}}+b$$ (4)

where *α* is the function’s maximal value, *k* represents its steepness at the midpoint *d_0_*, and *b* is an offset.

### Reduction to the Integrate-and-Fire point neuron model

All multicompartmental models examined in this work were computationally expensive, making the numerical simulation of large networks unfeasible. In the perspective of including an analysis based on the Liquid State Machine paradigm (see below), we reduced our multicompartmental models into equivalent eIF models [14], closely following the work of Gerstner and collaborators [22,23]. As a proof of principle, we restricted our efforts to the BAS models. Thus, for each soma-AIS distance d in the BAS model, a new set of eIF parameters was identified by fitting the eIF’s current-voltage relationship f(V_soma_), known analytically, to the current-voltage relationship of the BAS model, known numerically, while injecting the same noisy waveform in both models (i.e., I_1_ = 0, Fig 6):

$$f(V_{soma})=\frac{1}{\tau_{m}}(E_{L}-V_{soma}+\Delta_{T}e^{(V_{soma}-V_{T})/\Delta_{T}})$$ (5)

Thus, the eIF free parameters C_m_, τ_m_, E_L_, Δ_T_ and V_T_ could be set to best approximate the current-voltage relationship from the BAS simulations, measured as

$$f(V_{soma})=<\frac{d}{dt}V_{soma}-I/C_{m}>V_{soma}$$ (6)

where the average operator is applied for each value of the somatic voltage V_soma_. The model parameters obtained by the fitting procedure for eIF neurons are given in Table 3.

The remaining parameters were: membrane capacitance C_m_ = τ_m_, g_L_ = 14 pF, and the reset potential V_reset_ = -80 mV. The eIF also featured an absolute refractory period t_ref_ = 2 ms. We repeated the same optimization procedure for a set of different positions of the AIS along the axon, and each time we obtained a distinct set of eIF model parameters. In particular, such a systematic optimization resulted in a distinct set of values for the eIF parameter Δ_T_, which describes in eIF models the rapidity of the AP at its onset.

### Liquid State Machine: The classification task

We implemented a recurrent network of eIF model neurons, composed of 1000 excitatory and 250 inhibitory units (parameters are given in Table 4). Each neuron received current-based synaptic inputs from C_E_ presynaptic excitatory and C_I_ inhibitory neurons, randomly chosen. The network further received feed-forward inputs from a series of external units that projected to C_in_ randomly chosen excitatory neurons. The weights of all synaptic connections were drawn from a Gaussian random distribution with mean J* and standard deviation 0.7J*, except for feed-forward input synapses that were uniformly distributed in the range [0.5J_in_, 1.5J_in_]. Each neuron additionally received noisy background synaptic inputs, modeled implicitly as a Poisson process (rate: 20 event/s, J_noise_ = 2 nA). All excitatory synapses had propagation delays, drawn uniformly from [1, 10] ms for excitatory and from [0.1, 2] ms for inhibitory synapses. Synaptic transmission was modelled as additive currents, characterized by an instantaneous rise time and an exponential decay (time constant: 3 ms for excitatory inputs, 2 ms for inhibitory inputs). Finally, all recurrent connections experienced short-term depression and facilitation [20,33], where the efficacy of a synapse at the time of the n-*th* spike was determined by the base weight w_0_, a recovery state variable R_n_ and an utilization state variable u_n_ according to

$$w_{n}=w_{0}\;R_{n}\;u_{n}.$$ (7)

The recovery and the utilization terms *R*_*n*_ and *u*_*n*_ were updated each time a presynaptic spike occurred, using the following iterative expressions:

$$R_{1}=1$$ (8)

$$R_{n}=U+u_{n-1}(1-U)exp(-\frac{\Delta t}{F})$$ (9)

and

$$u_{1}=U$$ (10)

$$u_{n}=1+(R_{n-1}-R_{n-1}u_{n-1}-1)exp(-\frac{\Delta t}{D})$$ (11)

where Δt is the inter-spike interval, *U* is the utilization of synaptic resources for a single spike, and where *F* and *D* are the time constants for facilitation and depression processes, respectively. The parameters for each synapse type (Table 4) were chosen according to empirical data [34], as in [20].

**Table 4:** Connection parameters.

****connection****	*J* (*nA*)	*w*_0_ ****(nA)****	*U*	*D* (*ms*)	*F* (*ms*)	*Δ*_*syn*_ ****(ms)****	****deg.****	****C****
in	8.9	∼U(0.5J,1.5J)				∼U(1,10)	out-degree	70
EE	1.9	∼N(J,0.7J)	0.59	813	1	∼U(1,10)	in-degree	28
EI	6.7	∼N(J,0.7J)	0.049	399	1790	∼U(1,10)	in-degree	28
IE	6.1	∼N(J,0.7J)	0.016	45	376	∼U(0.1,2)	in-degree	48
II	4.9	∼N(J,0.7J)	0.25	706	21	∼U(0.1,2)	in-degree	48

N(μ,σ) denotes a normal distribution with mean *μ* and standard deviation *σ*. U(a,b) denotes a uniform distribution in [*a*,*b*]. The last column denotes whether connections are drawn with a fixed indegree or a fixed outdegree per neuron.

To obtain a well-performing network, we optimized the connectivity parameters (C_E_, C_I_, C_in_, see last column in Table 4) and the connection weight-means (J_EE_, J_EI_, J_IE_, J_II_, J_in_, second column in Table 4) using the eIF model corresponding to an AIS located at a distance of 25 μm from the soma (i.e. half of the range of considered values). We then investigated how the network performance changed when using eIF models fitted to models with different AIS locations.

The states of the network (i.e. the “liquid states”) were extracted from 200 randomly chosen excitatory neurons by filtering their AP trains with an exponential kernel (time constant of 20 ms). This extraction was repeated every 50 ms at the end of each epoch of external stimulation.

We trained the network to perform a delayed XOR on spike pre-defined templates. In each template presentation, 10 input units fired at predefined times (generated from a Poisson point process with a rate of 20 event/s) over an overall duration of 50 ms. The network was presented with jittered versions of these AP templates, where each input neuron activation time was shifted by a random number drawn from a Gaussian distribution with zero mean and standard deviation of 5 ms. These jittered templates were presented to the network in random order (Fig 7A–7C). We trained linear readout units on the network state to generate as output an XOR of the identity of the two last patterns.

The training of the readouts was carried out by running the network for 500 s, and then randomly splitting the sequence of resulting states into a training set (80%), used to train one linear classifier, and into a test set (20%), used for performance evaluation. For each run, we trained 100 classifiers for different random training/test splits, this allowed for a more robust performance estimation. We used the mean performance as the result of one run.

We then tested the LSM performance using all eIF models, i.e., we investigated the influence of AIS position on the network performance. As the performance of an LSM can significantly depend on the exact wiring (which is randomly drawn), we generated N = 50 networks (i.e. different randomly drawn connectivity, weights, and synaptic delays) and tested each eIF model on all such networks. This resulted in 50 performance values for each AIS position. For the efficiency of the LSM, we report the mean and standard deviations of these results. The p-values were computed using the Wilcoxon rank-sum test (Fig 7E).

We performed additional experiments to verify the robustness of these results (Figure C in S1 Text). In order to test whether the AP sharpness has a significant influence on network performance, we evaluated the network performance using the neuron parameters obtained via fitting at a soma-AIS distance of 25 μm while varying solely Δ_T_ in the value range found by the fitting method (see Results, Figure C, panel A, in S1 Text). In the reported simulations, we used longer delays for excitatory synapses in order to account for faster responses of inhibitory neurons. To test whether this choice influences our results, we repeated the complete experimental procedure (network parameter optimization and evaluation of network performance at different soma-AIS distances) using equal delays for excitatory and inhibitory connections (i.e., all synaptic delays were drawn from uniform distributions in [0.1, 2] ms). While this resulted in an overall decrease of performance, there was no qualitative change in the differential behavior for different soma-AIS distances (Figure C, panel B, in S1 Text). In our simulations, we optimized network parameters for an intermediate soma-AIS distance of 25 μm. In order to test whether this choice influences the results, we finally repeated the experimental procedure but optimized the network parameters for a soma-AIS distance of 0 μm, i.e., at a distance where we found the network to perform worst. The results were similar to those obtained by the optimization for a distance of 25 μm (Figure C, panel C, in S1 Text).

## Supporting information

S1 Text Three supplementary figures (Figure A, B, and C) display data and results from additional simulations.
(PDF)
