# Supplementary material for: The location of the axon initial segment affects the bandwidth of spike initiation dynamics
Source: PLoS Comput Biol. 2020 Jul 23;16(7):e1008087. doi: 10.1371/journal.pcbi.1008087 (PMC7402515; doi:10.1371/journal.pcbi.1008087)
Supplement: S1 Text — Three supplementary figures (Figure A, B, and C) display data and results from additional simulations. (PDF) [file pcbi.1008087.s001.pdf]

S1 – Text
The location of the axon initial segment affects the bandwidth of spike initiation dynamics

Christophe Verbist[1*], Michael G. Müller[2],
Huibert D. Mansvelder[3], Robert Legenstein[2], Michele Giugliano[1,4]

[1] Molecular, Cellular, and Network Excitability Laboratory, Institute Born-Bunge and Department of Biomedical Sciences, Universiteit Antwerpen, Wilrijk, Belgium
[2] Institute of Theoretical Computer Science, Graz University of Technology, Graz, Austria
[3] Department of Integrative Neurophysiology, Amsterdam Neuroscience, Center for Neurogenomics and Cognitive Research (CNCR), Vrije Universiteit Amsterdam, Amsterdam, The Netherlands
[4] Scuola Internazionale Superiore di Studi Avanzati, Neuroscience Area, Trieste, Italy

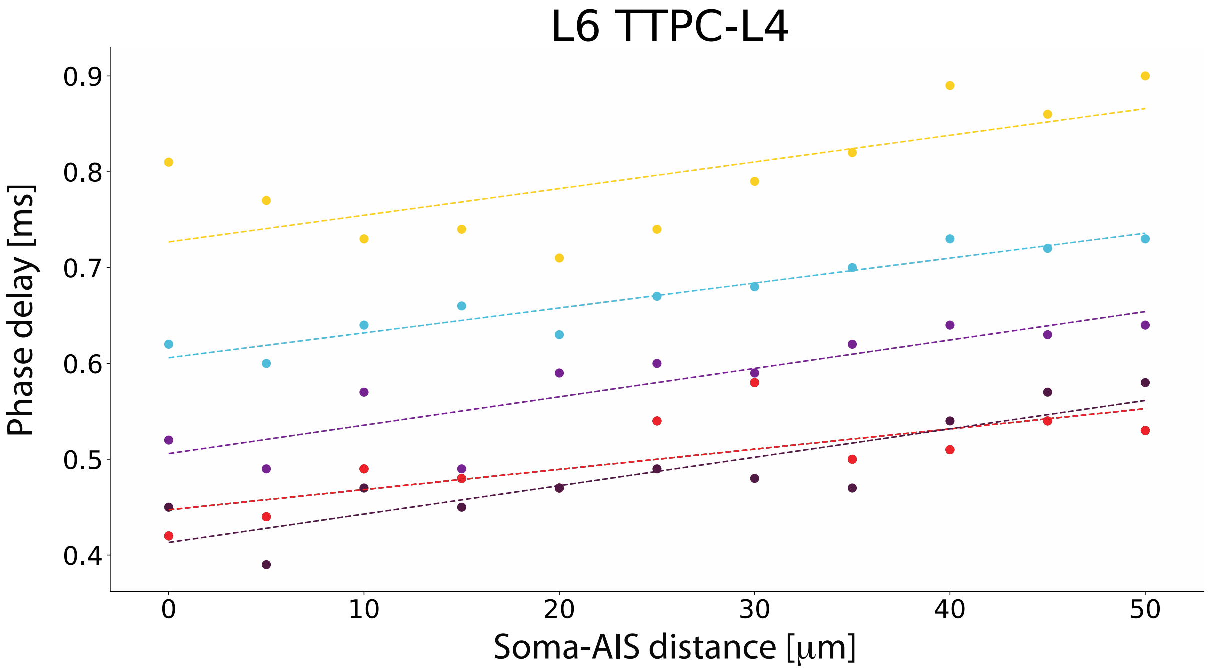

Figure A. AIS distance and AP propagation delays in model neurons. The phase of the dynamical transfer function of multicompartmental model neurons is characterized by a linear term in f, in the Fourier domain, for large harmonic frequencies. This corresponds, by definition of inverse Fourier, to a delay in the time domain. The figure displays the strong correlation of the phase delay with the AIS distance: the larger the AIS-soma distance is, the longer the delay for the AP to back propagate to the soma. The markers represent the time delay computed in the five “instances” (one for each color) of the same cell class (layer 4, TTPC), while the dashed lines represents their linear regressions.

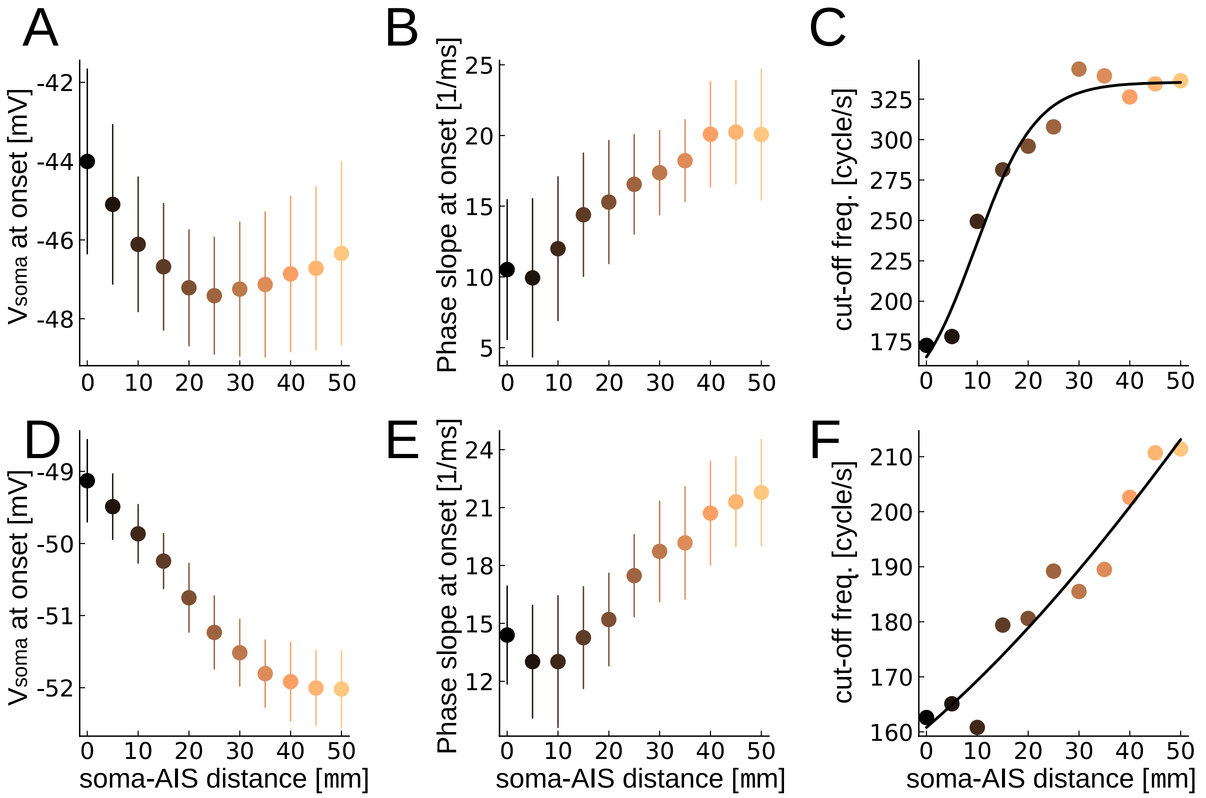

Figure B. The simulations of Fig. 3 were repeated after augmenting the axon model with 1 mm long unmyelinated (**A-C**) or myelinated (**D-F**) geometry extensions. Relocating the AIS up to 50 µm far from the soma had an impact on the AP initiation and its bandwidth qualitatively similar to Fig. 3: a decrease of the somatic membrane potential at the AP onset of a few mV (**A, D**), an increase in the phase slope at onset of ~10 units (**B, E**), and an increase in bandwidth (**C. F**). Quantitatively, the bandwidth varied for the same soma-AIS distance range for the unmyelinated axon (**C**) more than in Fig. 3 (i.e. +100% *vs* +63%) and for the myelinated axon (**F**) less than in Fig. 3 (i.e. +37% *vs* +63%). The continuous lines (**C**, **F**) represent the best fit logistic functions (eq. 4, as in Fig. 3F), whose parameters were $a = 203.95$, $k = 0.17$, $d_0 = 9.67$, and $b = 131.60$ (**C**) as well as $a = 257.79$, $k = 0.02$, $d_0 = 71.94$, and $b = 112.66$ (**F**).

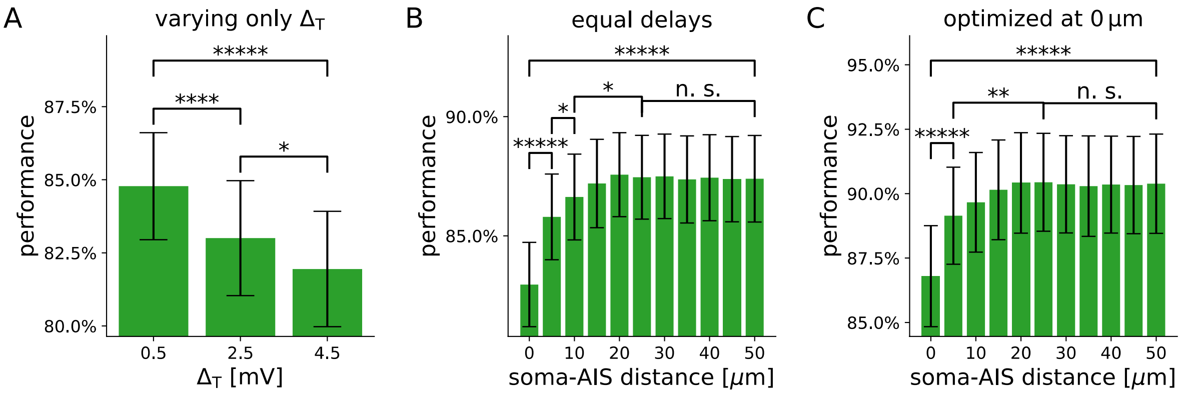

Figure C. Variations of the LSM experiment. (A) Performance when only Δ_T is varied. We repeated the LSM experiment (see Fig. 7) using the eIF parameters fit to an AIS-soma distance of 25 μm while varying the AP onset slope Δ_T in the range of the parameters fits (Table 3), using $\Delta_T \in \{0.5, 2.5, 4.5\}$ ms. Changing the AP slope led to significant changes of the LSM performance, demonstrating the importance of this parameter. (B) Performance when excitatory and inhibitory connections have similar delays. We changed the distribution of delays of the excitatory connections in the network (Table 4) to match those of the inhibitory connections (i.e. all synaptic delays drawn from uniform distributions in [0.1, 2] ms) and repeated the LSM experiment (optimization of network parameters and evaluation). Although there is a small overall decrease in performance, the results show similar behavior to the original experiment (Fig. 7) as the soma-AIS distance is increased. (C) Performance when network parameters are optimized for a different soma-AIS distance. We repeated the LSM experiment but optimized the network parameters for a soma-AIS distance of 0 μm. The results are very similar to the original experiment (Fig. 7).
